# State-Dependent Pulse Vaccination and Therapeutic Strategy in an SI Epidemic Model with Nonlinear Incidence Rate

**DOI:** 10.1155/2019/3859815

**Published:** 2019-02-06

**Authors:** Kaiyuan Liu, Tongqian Zhang, Lansun Chen

**Affiliations:** ^1^College of Mathematics and Information Science, Anshan Normal University, Anshan 114016, China; ^2^College of Mathematics and Systems Science, Shandong University of Science and Technology, Qingdao 266590, China; ^3^State Key Laboratory of Mining Disaster Prevention and Control Co-founded by Shandong Province and the Ministry of Science and Technology, Shandong University of Science and Technology, Qingdao 266590, China; ^4^Academy of Mathematics and Systems Science, Chinese Academy of Sciences, Beijing 100190, China

## Abstract

In this paper, the state-dependent pulse vaccination and therapeutic strategy are considered in the control of the disease. A pulse system is built to model this process based on an SI ordinary differential equation model. At first, for the system neglecting the impulse effect, we give the classification of singular points. Then for the pulse system, by using the theory of the semicontinuous dynamic system, the dynamics is analyzed. Our analysis shows that the pulse system exhibits rich dynamics and the system has a unique order-1 homoclinic cycle, and by choosing *p* as the control parameter, the order-1 homoclinic cycle disappears and bifurcates an orbitally asymptotical stable order-1 periodic solution when *p* changes. Numerical simulations by maple 18 are carried out to illustrate the theoretical results.

## 1. Introduction

Infectious diseases are caused by various pathogens that can be transmitted from person to person, animal to animal, or human to animal. The ever-changing changes in the pathogens of ancient infectious diseases and the emergence of new pathogens have brought new challenges to the discovery, diagnosis, and prevention of infectious diseases. According to the 2016 report of the World Health Organization [1], about 36.7 million people have been infected with HIV/AIDS, 1.0 million people died of HIV/AIDS, and more than 18 million people worldwide living with HIV are receiving antiviral drugs. And tuberculosis is currently the biggest “killer” caused by a single infectious pathogen after AIDS in the world. As of the end of 2016, there were 10.4 million new tuberculosis cases [2]. Therefore, the control and elimination of infectious diseases has attracted wide attention of people. Various dynamic models have been proposed by mathematicians to investigate the spread and evolution of infectious diseases [3–14]. In particular, mathematical models of differential equations have been extensively investigated, and among them, the most classical well-known model is SIR model [15] or SIS model [16], which have been widely investigated [17–23].

It is well known that vaccination is mostly a medical behavior that can evoke the individual's natural defense mechanism to prevent possible future diseases. This kind of vaccination is known as prophylactic vaccination. Diphtheria, whooping cough, polio, tetanus, herpes, rubella, and mumps are the most common types of vaccines. There are many types of vaccination, the two common types are continuous vaccination and pulsed vaccination. Continuous vaccination is when people are vaccinated at birth to protect themselves from illness, while pulsed vaccination is when people are vaccinated at a fixed period of time in all age groups which was firstly investigated by Agur et al in [24]. Pulse vaccination strategy (PVS) has been studied by many scholars [25, 26]. For example, Lu et al. [27] studied the pulse epidemic model with bilinear incidence and compared the effectiveness of the continuous and pulsed vaccination strategies. Liu et al. [28] investigated the SIR epidemic model with the saturated transmission rate. However, the strategy is taken at certain fixed times and does not depend on the status of infectious diseases. In general, taking into account the limited medical resources and costs, vaccines to susceptible people according to the number of susceptible people or infected people are more reasonable than continuous vaccination and fixed time pulse vaccination. This control strategy relies on the individual (or susceptible individuals) of the infection state and is called a state-dependent pulse vaccination strategy. Based on this idea, Tang et al. [29], Nie et al. [30], Guo et al. [31], and Qin et al. [32] have considered a state-dependent pulse strategy in SIR model and SIRS model. In fact, using state-dependent feedback control strategies to simulate real-world problems is more reasonable. Therefore, the impulsive state feedback control is also widely applied to the population dynamics model [33–46], chemostat model [47], and turbidostat model [48].

Firstly, we consider an SI epidemic model with nonlinear incidence rate *βSI*^2^ described by the ordinary differential equations as follows:

(1) $$\begin{array}{c} \left\{\begin{array}{c} \frac{dS}{dt}=\theta -\beta SI^{2}-\gamma S,\\ \frac{dI}{dt}=\beta SI^{2}-\gamma I, \end{array}\right. \end{array}$$

which is a special case in the study of Liu et al. [49] and *S*(*t*) and *I*(*t*) represents the number of susceptible and infected individuals at time *t*, respectively. *θ* is the birth rate, *β* is the contact rate, and *γ* is the natural death rate.

Motivated by the studies of Tang et al. [29], Nie et al. [30], and Zhang et al. [50], we consider state-dependent pulse vaccination and treatment strategy in model (1) and get the following model:

(2) $$\begin{array}{c} \left\{\begin{array}{c} \left.\begin{array}{c} \frac{dS}{dt}=\theta -\beta SI^{2}-\gamma S,\\ \frac{dI}{dt}=\beta SI^{2}-\gamma I, \end{array}\right\}\;I<h_{1},\\ \left.\begin{array}{c} \Delta S=-pS,\\ \Delta I=-qI, \end{array}\right\}\;I<h_{1}, \end{array}\right. \end{array}$$

where Δ*S*(*t*)=*S*(*t*^+^) − *S*(*t*)  and  Δ*I*(*t*)=*I*(*t*^+^) − *I*(*t*). When the amount of infected reaches the hazardous threshold value *h*_1_(>0), vaccination and treatment are taken into account, and the number of susceptible and infected suddenly turn to (1 − *p*)*S*(*t*) and (1 − *q*)*I*(*t*), respectively, where 0 < *p*  and  *q* < 1 denote the vaccination rate of susceptible individuals and treatment rate of infected individuals, respectively. By the scaling,

(3) $$\begin{array}{c} S=k_{1}x,\\ I=k_{2}y,\\ t=k_{3}\tau ,\\ \frac{k_{3}}{k_{1}}\theta =a,\\ \beta k_{2}^{2}k_{3}=1,\\ k_{3}=\frac{1}{\gamma }, \end{array}$$

then, model (2) transforms into the following form:

(4) $$\begin{array}{c} \left\{\begin{array}{c} \left.\begin{array}{c} \frac{dx}{d\tau }=a-y^{2}x-x,\\ \frac{dy}{d\tau }=y^{2}x-y, \end{array}\right\}\;\Delta y=-qy,\\ \left.\begin{array}{c} \Delta x=-px,\\ \Delta y=-qy, \end{array}\right\}\;y=h_{2}, \end{array}\right. \end{array}$$

where *h*_2_=*h*_1_/*k*_2_. In the following, according to the actual condition, we always suppose that *h* ≤ *y*_2_, and based on practical significance, our research scope is limited to the first quadrant, i.e., *R*_+_^2^={(*x*, *y*)|*x* ≥ 0, *y* ≥ 0}.

The purpose of this paper is to study the dynamic behavior under the effect of state-dependent pulse vaccination and treatment strategy. This article is organized as follows. In Section 2, we introduce some definitions and notations of the geometric theory of semicontinuous dynamic systems, which will be useful for the latter discussion. In Section 3, we qualitatively analyze the dynamics of model (3). In Section 4, the existence of the homoclinic cycle is studied by using the geometrical theory of semicontinuous dynamical systems. At last, we present some numerical simulations.

## 2. Preliminaries

In this section, we introduce some notations, definitions, and lemmas of the geometric theory of semicontinuous dynamic system, which will be useful for the following discussions. The following definitions and lemmas of semicontinuous dynamic system come from the studies of Chen et al. [51] and Wei and Chen [36].

**Definition 1 .**

Consider the following state-dependent impulsive differential system

(5) $$\begin{array}{c} \left\{\begin{array}{c} \frac{dx}{dt}=P\left(x,y\right),\;\frac{dy}{dt}=Q\left(x,y\right),\;\left(x,y\right)\notin M\left\{x,y\right\},\\ \Delta x=\alpha \left(x,y\right),\;\Delta y=\beta \left(x,y\right),\;\left(x,y\right)\in M\left\{x,y\right\}. \end{array}\right. \end{array}$$

The solution mapping of system (4) is called the semicontinuous dynamical system denoted by Ω, *f*, *φ*,  and *M*, where (*x*, *y*) ∈ Ω⊂*R*_+_^2^  and  *f*=*f*(*p*; *t*) is the semicontinuous dynamical system mapping with initial point *p*=(*x*_0_, *y*_0_) ∉ *M*; the sets *M* and *N* are called the impulse set and phase set, which are lines or curves on *R*_+_^2^. The continuous function *φ* : *M*⟶*N* is called impulse mapping.

**Remark 1 .**

System (4) constitutes a semicontinuous dynamic system (Ω, *f*, *φ*, *M*), where Ω=*R*_+_^2^={(*x*, *y*)|*x* ≥ 0, *y* ≥ 0}, *M*={(*x*, *y*) ∈ *R*_+_^2^|*x* ≥ 0, *y*=*h*_2_}, *φ* : (*x*, *y*) ∈ *M*⟶((1 − *p*)*x*, (1 − *q*)*h*_2_) ∈ *R*_+_^2^, *N*=*φ*(*M*)={(*x*, *y*) ∈ *R*_+_^2^|*x* ≥ 0, *y*=(1 − *q*)*h*_2_}.

**Definition 2 .**

If there exists a point *P* ∈ *N* and *T* > 0 such that *f*(*P*, *T*)=*Q* ∈ *M* and *φ*(*Q*)=*φ*(*f*(*P*, *T*))=*P* ∈ *N*, then *f*(*P*, *t*) is called order-1 periodic solution.

**Definition 3 .**

The trajectory *f*(*P*, *t*) combining with impulse line QP is called the order-1 cycle. If the order-1 cycle has a singularity, then the order-1 cycle is called the order-1 singular cycle. Furthermore, if the order-1 cycle only has a saddle, then the order-1 singular cycle is called the order-1 homoclinic cycle.

**Definition 4 .**

We assume that G is a bounded closed simple connected region, which has the following properties:

- Impulse set *M* is a simple connected bounded closed straight line segments or curve segments which do not contain closed branch
- The boundaries AD, BC, and AB of region G are nontangent arcs of semicontinuous dynamical system (4). The boundary CD is the impulse set of system (4), and its phase set satisfies *φ*(CD) ⊆ AB;
- The orientation of the vector fields of semicontinuous dynamical system (4) on the AD, BC, and AB points of the internal of region G. There are no equilibriums on the boundaries and also in the internal of region G of semicontinuous dynamical system (4).

Then region G is called Bendixson's region of semicontinuous dynamical system (4).

**Figure 1:**
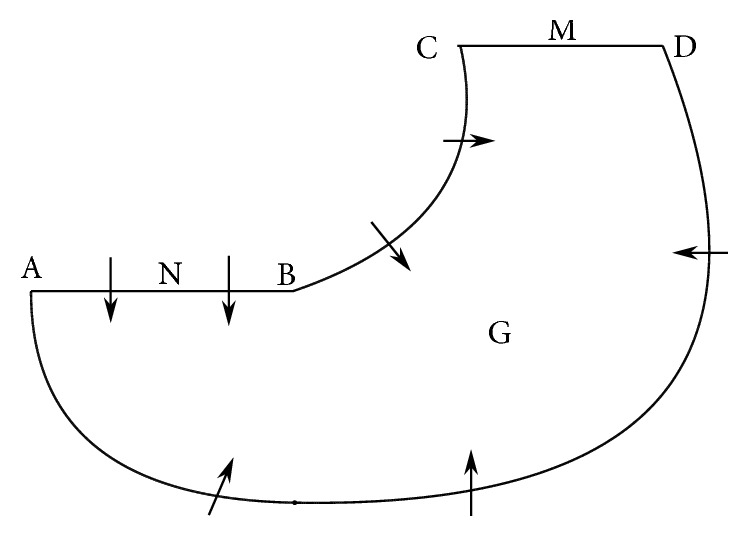
Bendixon region of the semicontinuous dynamical system. This figure is reproduced from the study of Wei and Chen [36] (under the Creative Commons Attribution License/public domain).

**Lemma 1 .**

(Bendixson theorem of semicontinuous dynamical system.) If region G is Bendixson's region of semicontinuous dynamical system (4), then there exists at least an order-1 periodic solution in the internal of region G (Figure 1).

Next, we will give the definition of successor function of system (4). Firstly, we define a new number axis in set *N*. On straight line *y*=(1 − *q*)*h*_2_, take the origin at point (0, (1 − *q*)*h*_2_) of coordinate axis *y* and define the positive direction and unit length to be consistent with coordinate axis *x*, then we obtain a number axis *l*. For any *A* ∈ *l*, let *l*(*A*) be the coordinate of point *A* which is defined as the distance between point *A* and the *y*-axis, i.e., *l*(*A*)=*x*_A_.

**Definition 5 .**

**Figure 2:**
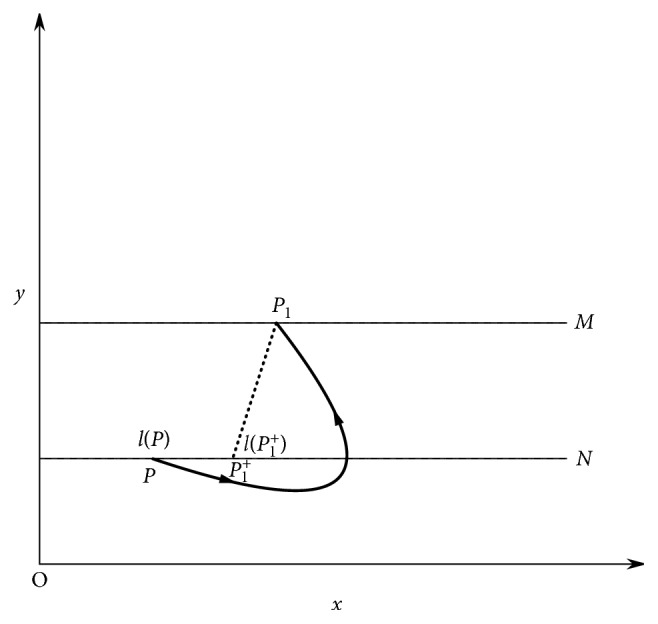
Successor function.

Suppose *g* : *N*⟶*N* be a map. Let *P* ∈ *N* be the initial mapping point, for any *P* ∈ *N*, there exists a *t*_1_ > 0 such that *F*(*P*)=*f*(*P*, *t*_1_)=*P*_1_ ∈ *M*, *P*_1_^+^=*φ*(*P*_1_) ∈ *N*. Then, function *g*(*P*)=*l*(*P*_1_^+^) − *l*(*P*) is the successor function of point *P*, and the point *P*_1_^+^ is called the successor point of *P* (Figure 2).

**Definition 6 .**

Suppose Γ=*f*(*P*, *t*) is an order-1 periodic solution of system (4). If for any *ε* > 0, there must exist *δ* > 0 and *t*_0_ ≥ 0 such that, for any point *P*_1_ ∈   ∪  (*P*, *δ*) ∩ *N*, we have *ρ*(*f*(*P*_1_, *t*), Γ) < *ε* for *t* > *t*_0_; then we call the order-1 periodic solution Γ is orbitally asymptotically stable.

## 3. Qualitative Analysis of System Neglecting the Impulse Effect

First, we consider the classification of singular points of the system neglecting the impulse effect. Neglecting the impulse effects, system (3) reduces to

(6) $$\begin{array}{c} \left\{\begin{array}{c} \frac{dx}{d\tau }=a-y^{2}x-x,\\ \frac{dy}{d\tau }=y^{2}x-y, \end{array}\right. \end{array}$$

system (5) always has one equilibrium *E*_0_(*a*, 0). Denote *R*_0_=*a*^2^/4; if *R*_0_ > 1, the system (5) has two positive equilibria *E*_1_(*x*_1_, *y*_1_)  and  *E*_2_(*x*_2_, *y*_2_), where $x_{1}=\left(a+\sqrt{a^{2}-4}/2\right),\;\;y_{1}=\left(a-\sqrt{a^{2}-4}/2\right)$ and $x_{2}=\left(a-\sqrt{a^{2}-4}/2\right),y_{2}=\left(a+\sqrt{a^{2}-4}/2\right)$.

Next, we will analysis of the stability of the equilibria of system (5). For equilibrium *E*_0_, we have

(7) $$\begin{array}{c} J_{E_{0}}=\left(\begin{array}{cc} -1 & 0\\ 0 & -1 \end{array}\right), \end{array}$$

obviously *E*_0_ is a stable node.

For *E*_1_, we have

(8) $$\begin{array}{c} J_{E_{1}}=\left(\begin{array}{cc} -1-y_{1}^{2} & -2x_{1}y_{1}\\ y_{1}^{2} & -1+2x_{1}y_{1} \end{array}\right), \end{array}$$

and the characteristic equation is *λ*^2^+*y*_1_^2^*λ*+*y*_1_^2^ − 1=0. Let *λ*_1_ and *λ*_2_ be the two characteristic roots of the characteristic equation, then we have

(9) $$\begin{array}{c} \lambda_{1}+\lambda_{2}=-y_{1}^{2}<0,\\ \lambda_{1}\lambda_{2}=y_{1}^{2}-1=\frac{y_{1}}{x_{1}}-1=\frac{-2\sqrt{a^{2}-4}}{a+\sqrt{a^{2}-4}}<0, \end{array}$$

obviously *E*_1_ is a saddle.

**Figure 3:**
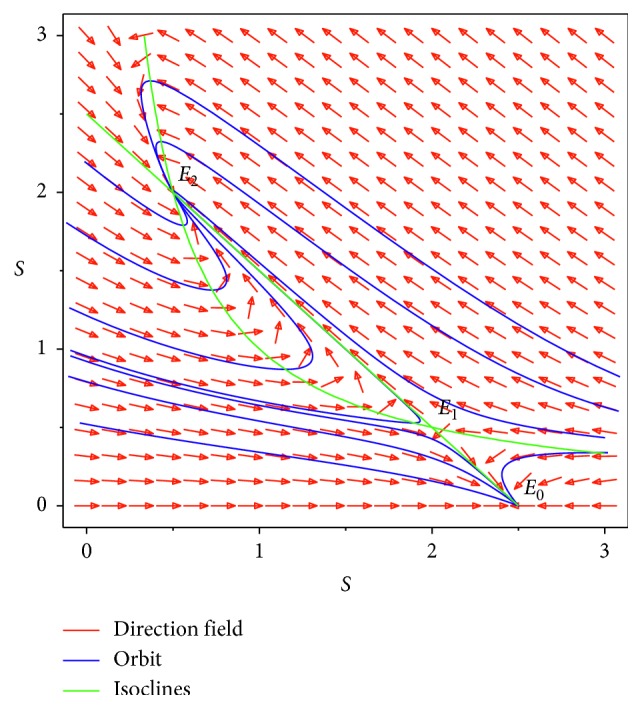
Phase diagram of system (3) with *θ*=2.5, *β*=1, and  *γ*=1.

For *E*_2_, we have

(10) $$\begin{array}{c} J_{E_{2}}=\left(\begin{array}{cc} -1-y_{2}^{2} & -2x_{2}y_{2}\\ y_{2}^{2} & -1+2x_{2}y_{2} \end{array}\right), \end{array}$$

and by calculations, we get

(11) $$\begin{array}{c} \lambda_{1}+\lambda_{2}=-y_{2}^{2}<0,\\ \lambda_{1}\lambda_{2}=y_{2}^{2}-1=\frac{y_{2}}{x_{2}}-1=\frac{2\sqrt{a^{2}-4}}{a-\sqrt{a^{2}-4}}>0, \end{array}$$

obviously *E*_2_ is a stable node (Figure 3).

**Lemma 2 .**

System (5) is uniformly bounded.

**Proof**

**Figure 4:**
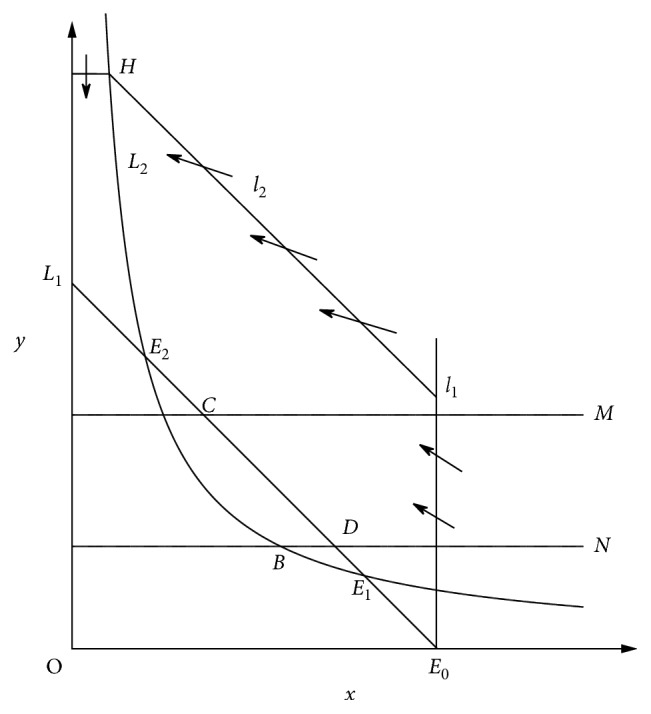
System (3) is the uniformly bounded.

Firstly, if we have the isoclines *L*_1_ : *dx*/*dt*=0 and *L*_2_ : *dy*/*dt*=0 (Figure 4) and the straight line *l*_1_ : *x* − *a*=0, then we get *dl*_1_/*dt*=*dx*/*dt|*_*x*=*a*_=−*ay*^2^ < 0; thus, according to the qualitative theory of ordinary differential equations, the trajectory of the system (5) passes through *l*_1_ and goes from the right side of *l*_1_ to the left side of *l*_1_. Consider the straight line *l*_2_ : *x*+*y* − *M*=0, where *M* is large enough and 0 ≤ *x* ≤ *a*. Then, we obtain *dl*_2_/*dt|*_*l*_2_=0_=*a* − *M* < 0, and thus, the straight line *l*_2_ is nontangent; then, according to the qualitative theory of ordinary differential equations, the trajectory of the system (5) passes through *l*_2_ and goes from the upper right side of *l*_2_ to the lower left side of *l*_2_. Let us denote the intersections of *l*_2_ and *L*_2_ be *H*(*x*_*H*_, *y*_*H*_) and consider the straight line *l*_3_ : *y* − *y*_*H*_=0, obviously, we have *dy*/*dt|*_*l*_3_=0_ < 0, and then, the trajectory of model (5) passes through *l*_3_ and goes from the top side of *l*_3_ to the bottom side of *l*_3_. Thus, the model (5) is uniformly upper bounded. This completes the proof.

## 4. Homoclinic Cycle of Model about Parameter *p*

In this section, we will discuss the existence of order-1 homoclinic cycle of model (3) by choosing *p* as the control parameter.

**Theorem 1 .**

If *R*_0_ > 1, then there exists *p*′ ∈ (0,1) such that model (3) has an order-1 homoclinic cycle.

**Proof**

**Figure 5:**
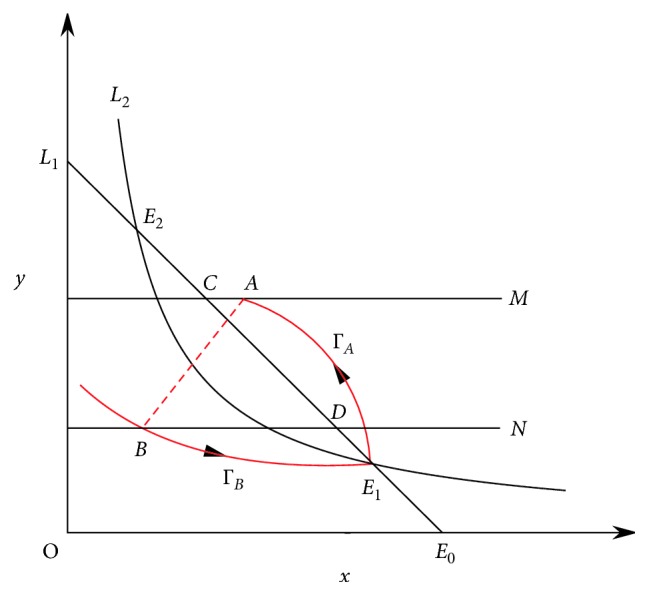
The existence of the order-1 homoclinic cycle.

In model (3), since the point *E*_1_ is a saddle point, then there exist two manifolds which will enter or leave the saddle point *E*_1_, one is the unstable manifold (Γ_*A*_) and another is the stable manifold Γ_*B*_. According to Lemma 2 and the qualitative theory of ordinary differential equations, Γ_*A*_ and impulse set *M* must intersect, and the intersection is denoted as *A*(*x*_*A*_, *y*_*A*_). If we denote the intersection of impulse set *M* and the isocline *L*_1_ as point *C*(*x*_*C*_, *y*_*C*_), the intersection of image set *N* and isoclines *L*_1_ as point *D*(*x*_*D*_, *y*_*D*_), and the intersection of image set *N* and Γ_*B*_ as point *B*(*x*_*B*_, *y*_*B*_), by the qualitative theory of ordinary differential equations, the unstable manifold Γ_*A*_ is above of the isoclines *L*_1_, and the stable manifold Γ_*B*_ is below the isoclines *L*_2_(*dy*/*dt*=0) (Figure 5). Because the monotonicity of the impulse function *φ*(*x*, *p*)=(1 − *p*)*x* with respect to *x* and *p*, there must exist *p*′ ∈ (0,1) such that *φ*(*x*_A_, *p*′)=(1 − *p*′)*x*_A_=*x*_B_, and then the stable manifold Γ_*B*_ starting form the point *B*, the unstable manifold Γ_*A*_ starting form the point *E*_1_, and the impulse line AB formed a homoclinic cycle.

**Remark 2 .**

If *p* > *p*′, according to the theory of differential equations, the trajectory tends to *E*_0_, and in a biological sense, the disease eventually extincts. However, the relatively high vaccination rate will waste medical resources. So, we always assume that *p* < *p*′ in the following theorem.

**Theorem 2 .**

If *R*_0_ > 1, *p* < *p*′, and *p*′ − *p* ≪ 1, then the homoclinic cycle of model (3) disappears and bifurcates an unique order-1 periodic solution.

**Proof**

**Figure 6:**
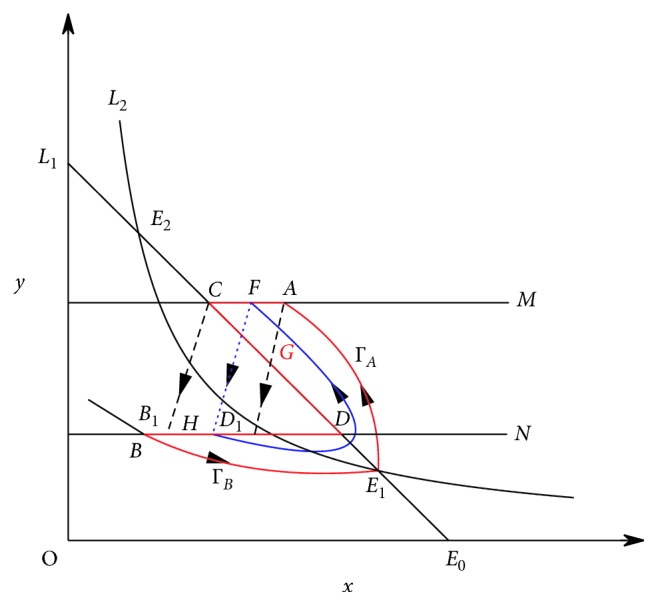
The existence of order-1 periodic solution of system (3).

By Theorem 1, if *R*_0_ > 1, then there exists *p*′ ∈ (0,1) such that model (3) has an order-1 homoclinic cycle, i.e., the stable manifold Γ_*B*_ starting form the point *B*, the unstable manifold Γ_*A*_ starting form the point *E*_1_, and the impulse line AB formed a homoclinic cycle. Now, we consider whether there will be a periodic solution that bifurcates out of the homoclinic cycle when *p* changes. In fact, consider the unstable manifold Γ_*A*_ starting form the point *E*_1_, when Γ_*A*_ touches the impulsive set *M* (the intersection is denoted as *A*), then a pulse happens and then the impulsive function transfers the point *A* into *D*_1_ and the point *C* into *B*_1_, and according the definition of impulsive function, we have *φ*(*x*_*A*_, *p*)=(1 − *p*)*x*_*A*_=*x*_*D*_1__, *φ*(*x*_*C*_, *p*)=(1 − *p*)*x*_*C*_=*x*_*B*_1__. If *p* < *p*′, we obtain *x*_*D*_1__ > *x*_*B*_. Since *x*_*B*_ ≤ *φ*(*x*_*C*_, *p*)=*x*_*B*_1__  and  *x*_*D*_ ≥ *φ*(*x*_*A*_, *p*)=*x*_*D*_1__, then we get *x*_*D*_ ≥ *x*_*D*_1__ ≥ *x*_*B*_1__ ≥ *x*_*B*_, and then by the definition of Bendixon region of semicontinuous dynamics system, *AC*, *CD* (part of isoclinal *L*_1_), *DB* (*B*_1_*D*_1_ ⊂ *BD*), *BE*_1_ (part of the Γ_*B*_), and *E*_1_*A* (part of the Γ_*A*_) constitute the Bendixon region G of the system (3). According to Lemma 1 and Lemma 2 in [36], system (3) must exist an order-1 periodic solution, initial point of which is between *B*_1_ and *D*_1_ in image set *N* (Figure 6).

**Figure 7:**
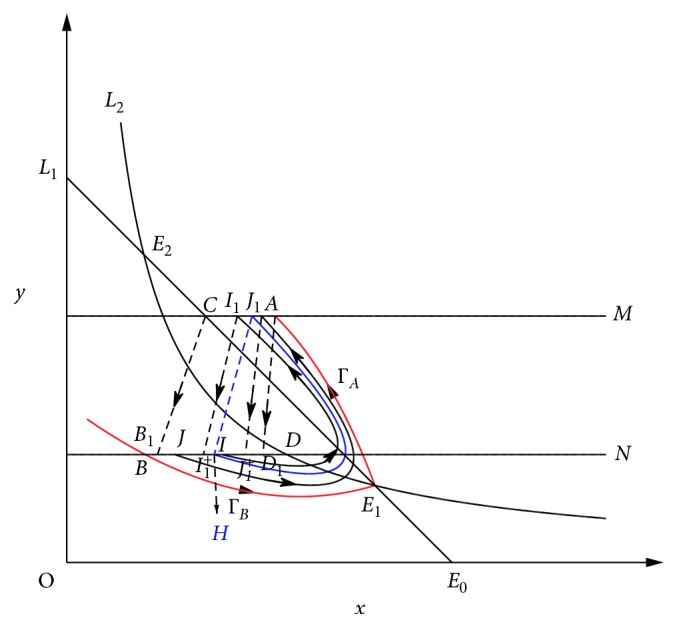
The monotonicity of the successor function G in the segment *A*_1_*B*_1_.

Next, we show the order-1 periodic solution of system (3) is unique if it exists. The idea of the proof comes from the study of Wei and Chen [36]. Select two points *I* and *J* in phase set *B*_1_*D*_1_ arbitrarily, where *x*_*B*_1__ < *x*_*J*_ < *x*_*I*_ < *x*_*D*_1__. Let *F*(*I*)=*I*_1_ ∈ *M*  and  *F*(*J*)=*J*_1_ ∈ *M*, after that due to the impulsive effects, points *I*_1_ and *J*_1_ jump to *I*_1_^+^, *J*_1_^+^ ∈ *N*. For *x*_*J*_ < *x*_*I*_, we have *x*_*I*_1__ < *x*_*J*_1__ and *x*_*I*_1_^+^_=(1 − *p*)*x*_*I*_1__, *x*_*J*_1_^+^_=(1 − *p*)*x*_*J*_1__; hence, we have *x*_*I*_1_^+^_ < *x*_*J*_1_^+^_. Using Definition 5 and Definition 6 in [36], we obtain *g*(*I*)=*x*_*I*_1_^+^_ − *x*_*I*_ and *g*(*J*)=*x*_*J*_1_^+^_ − *x*_*J*_. Hence, we have *g*(*I*) − *g*(*J*)=(*x*_*I*_1_^+^_ − *x*_*I*_) − (*x*_*J*_1_^+^_ − *x*_*J*_)=(*x*_*J*_ − *x*_*I*_)+(*x*_*I*_1_^+^_ − *x*_*J*_1_^+^_) < 0; that is, the successor function *g*(*p*) is monotonic in *B*_1_*D*_1_. Therefore, there is an unique point *H* such that *g*(*H*)=0; thus, system (3) has an unique order-1 periodic solution (Figure 7).

**Theorem 3 .**

If *R*_0_ > 1, *p* < *p*′, and *p*′ − *p* ≪ 1, then the order-1 periodic solution of model (3) is orbitally asymptotically stable.

**Proof**

**Figure 8:**
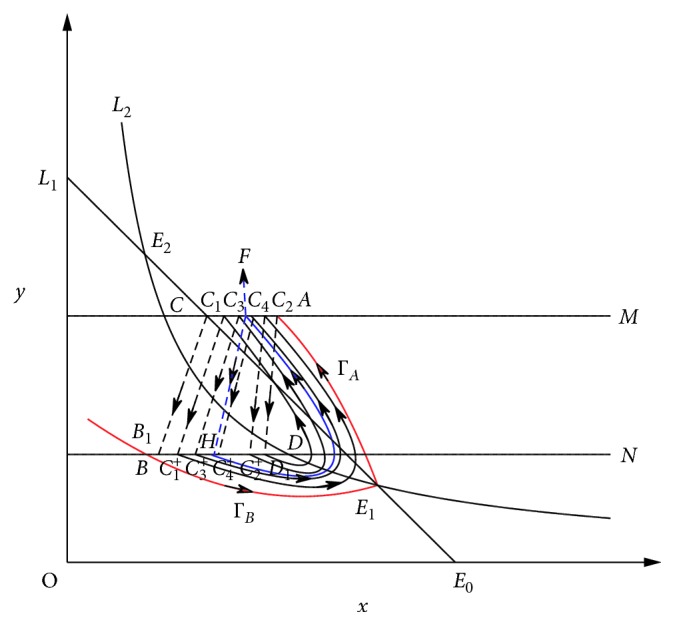
The stability of order-1 periodic solution of system (3).

By Theorem 2, we have that the order-1 periodic solution in system (3) is unique. Let the initial point of the order-1 periodic solution is *H* ∈ *B*_1_*D*_1_, where *x*_*B*_1__ < *x*_*H*_ < *x*_*D*_1__. Set *F*(*D*_1_)=*C*_1_ ∈ *M*, then due to the impulsive effects *C*_1_ jumps to *C*_1_^+^ which is the successor point of *D*_1_ (Figure 8). We have *x*_*B*_1__ < *x*_*C*_1_^+^_ < *x*_*H*_. Set *F*(*C*_1_^+^)=*C*_2_ ∈ *M*. Owing to trajectories do not intersect, we have *x*_*H*_ < *x*_*C*_2_^+^_ < *x*_*D*_1__ and *x*_*C*_1__ < *x*_*F*_ < *x*_*C*_2__ < *x*_*A*_, where *F* is the impulsive point of the order-1 periodic solution.

Similar to the above method, let *F*(*C*_2_^+^)=*C*_3_ ∈ *M*, we have *x*_*C*_1_^+^_ < *x*_*C*_3_^+^_ < *x*_*H*_ < *x*_*C*_2_^+^_ and *x*_*C*_1__ < *x*_*C*_3__ < *x*_*F*_ < *x*_*C*_2__. We can repeat the above steps and have a sequence {*C*_*k*_}_*k*=1,2,…_ of impulse set *M* and a sequence {*C*_*k*_^+^}_*k*=1,2,…_ of image set *N* satisfying *F*(*C*_*k*_^+^)=*C*_*k*+1_, *x*_*C*_2*k*−1_^+^_ < *x*_*C*_2*k*+1_^+^_ < *x*_*H*_ < *x*_*C*_2*k*_^+^_, *x*_*C*_2*k*−1_^+^_ < *x*_*H*_ < *x*_*C*_2*k*_^+^_ < *x*_*C*_2*k*−2_^+^_. That is, we have

(12) $$\begin{array}{c} x_{B_{1}}<x_{C_{1}^{+}}<x_{C_{3}^{+}}<\cdots <x_{C_{2k-1}^{+}}<x_{C_{2k+1}^{+}}<\cdots <x_{H},\\ x_{D_{1}}>x_{C_{2}^{+}}>x_{C_{4}^{+}}>\cdots >x_{C_{2k}^{+}}>x_{C_{2\left(k+1\right)}^{+}}>\cdots >x_{H}. \end{array}$$

Thus, series {*x*_*C*_2*k*−1_^+^_}, *k*=1,2,…, is monotonically increasing, and {*x*_*C*_2*k*_^+^_}, *k*=1,2,…, is monotonically decreasing; *x*_*C*_2*k*_^+^_⟶*x*_*H*_, as *k*⟶*∞*, and *x*_*C*_2*k*−1_^+^_⟶*x*_*H*_, as *k*⟶*∞*. Select a point *H*_0_ ∈ *C*_1_^+^*D*_1_ that is different from point *H*. If *x*_*H*_ < *x*_*H*_0__ < *x*_*D*_1__ (otherwise, *x*_*C*_1_^+^_ < *x*_*H*_0__ < *x*_*H*_, the discussion is similar to *x*_*H*_ < *x*_*H*_0__ < *x*_*D*_1__), there must be an integer *k* such that *x*_*C*_2(*k*+1)_^+^_ < *x*_*H*_0__ < *x*_*C*_2*k*_^+^_. The orbit starting from point *H*_0_ will also experience an infinite number of impulsive effects. Let the phase point be *H*_*l*_, *l*=0,1,2,… which is after the *l*^th^ impulsive effect. Then for any *l*, we have *x*_*C*_2(*k*+*l*+1)_^+^_ < *x*_*H*_2*l*__ < *x*_*C*_2(*k*+*l*)_^+^_ and *x*_*C*_2(*k*+*l*)+1_^+^_ < *x*_*H*_2*l*+1__ < *x*_*C*_2(*k*+*l*+1)+1_^+^_. Hence, {*x*_*H*_2*l*__}, *l*=0,1,2,…, is monotonically decreasing, and {*x*_*H*_2*l*+1__}, *i*=0,1,2,…, is monotonically increasing. Thus, after the pulse effects the successor points are attracted to the point *H*, which means that the order-1 periodic solution of the system (3) is orbitally asymptotically stable.

## 5. Numerical Simulations

In this section, we give some numerical simulations to illustrate the theoretical results we previously obtained. First, we consider the system neglecting state-dependent pulse strategy, let *θ*=2.5, *β*=1, *γ*=1,  and *h*=1.6, and simple calculations show *R*_0_=1.5625; then system (3) has three equilibria, i.e., *E*_0_=(2.5, 0), *E*_1_=(2,0.5), and *E*_2_=(0.5, 2) (Figure 3), and among them, *E*_0_ is a stable node, *E*_1_ is a saddle, and *E*_2_ is a stable node.

**Figure 9:**
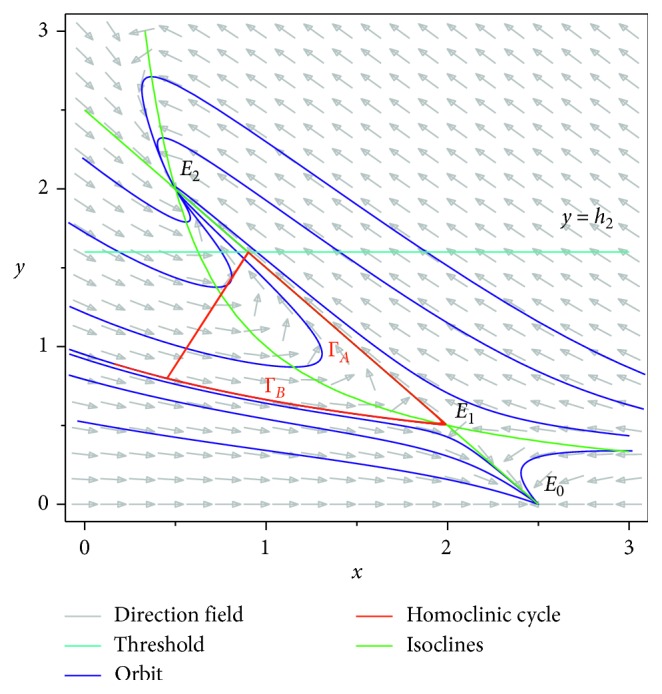
Order-1 homoclinic cycle of system (3) with *p*=0.496  and  *q*=0.5.

**Figure 10:**
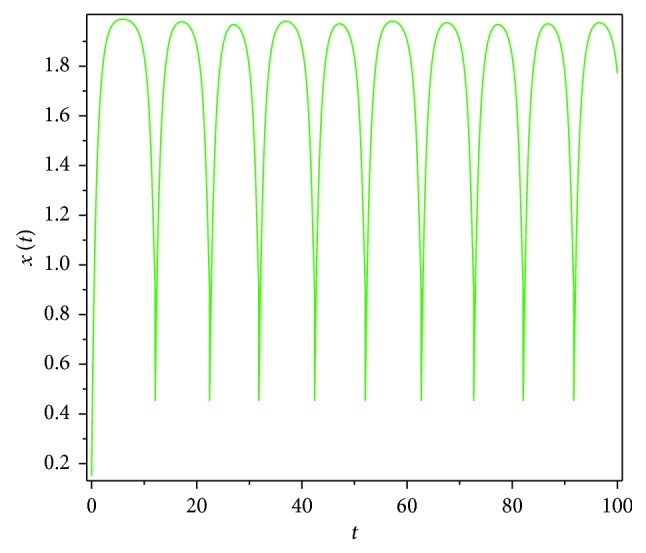
Time series diagram of *x*(*t*) of system (3) with *p*=0.496  and  *q*=0.5.

**Figure 11:**
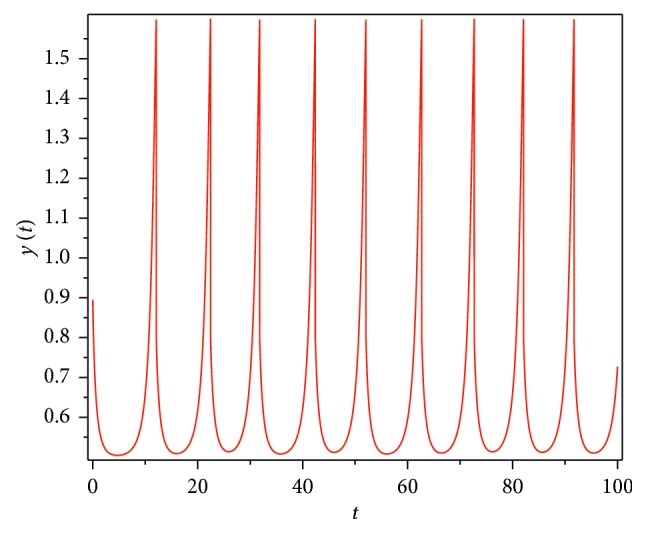
Time series diagram of *y*(*t*) of system (3) with *p*=0.496  and  *q*=0.5.

**Figure 12:**
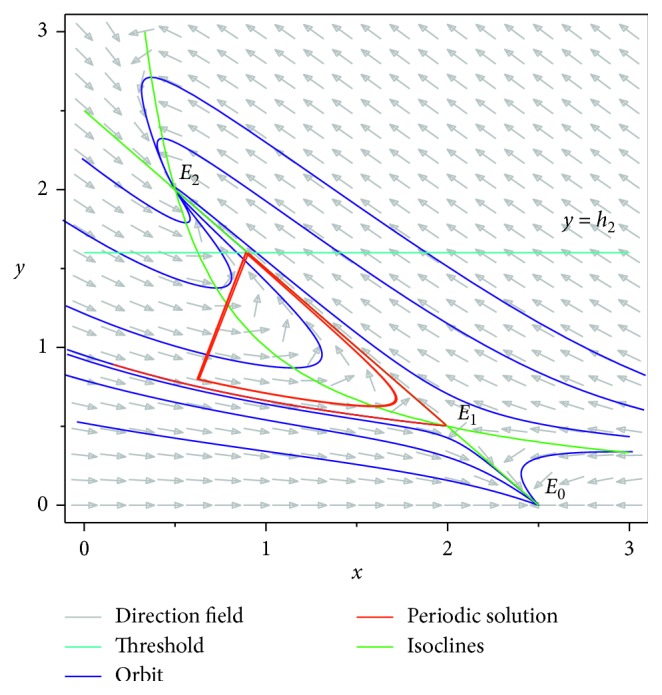
Order-1 homoclinic bifurcation of system (3) with *p*=0.3.

**Figure 13:**
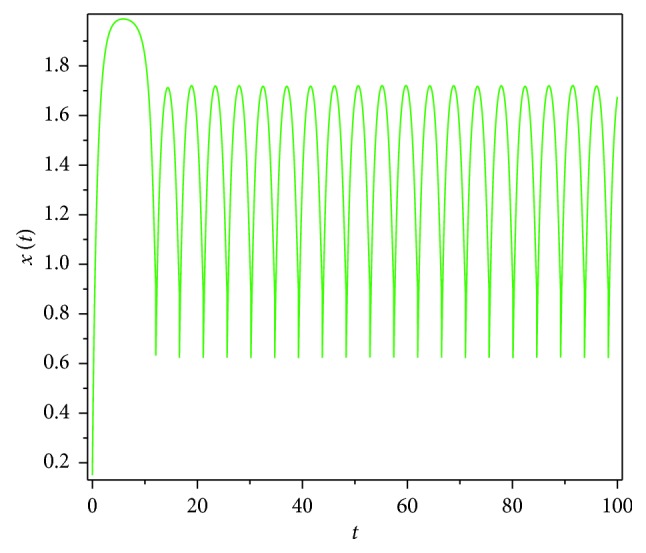
Time series diagram of *x*(*t*) of system (3) with *p*=0.3.

**Figure 14:**
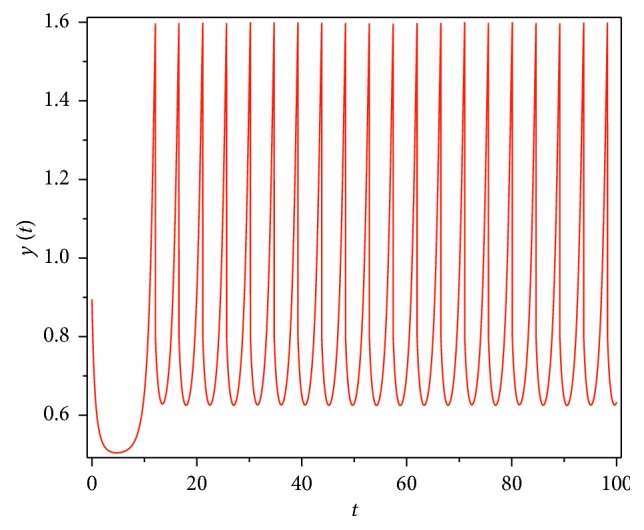
Time series diagram of *y*(*t*) of system (3) with *p*=0.3.

**Figure 15:**
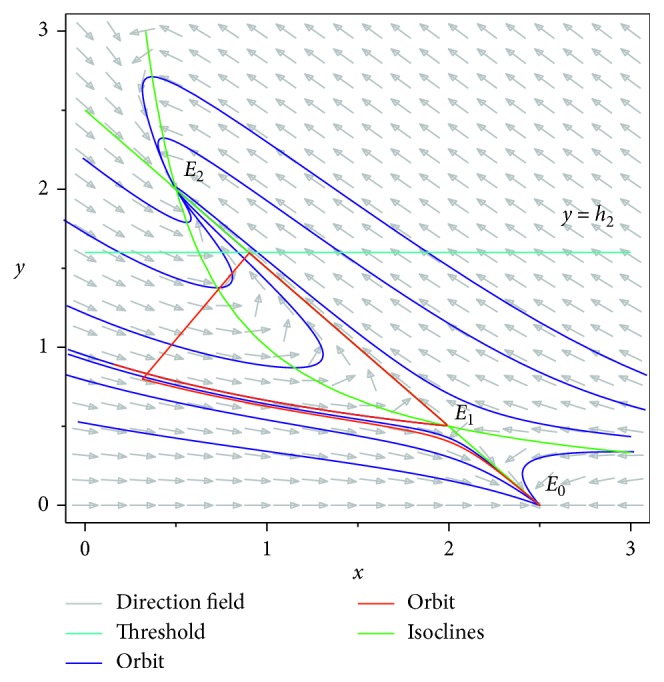
Phase diagram of system (3) with *p*=0.65.

**Figure 16:**
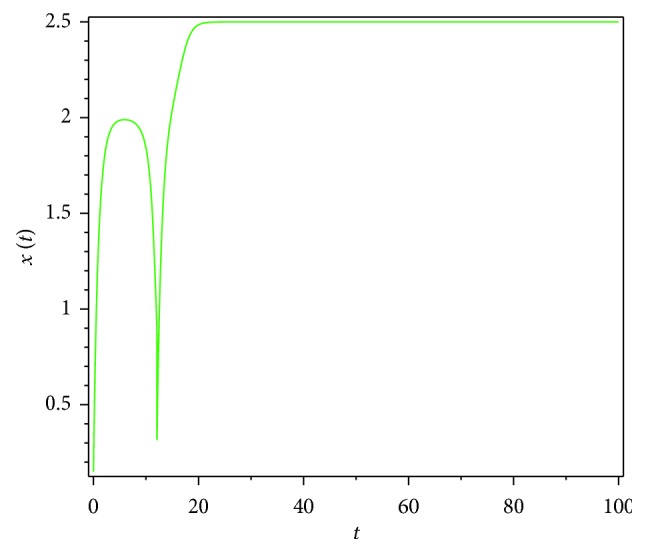
Time series diagram of *x*(*t*) of system (3) with *p*=0.65.

**Figure 17:**
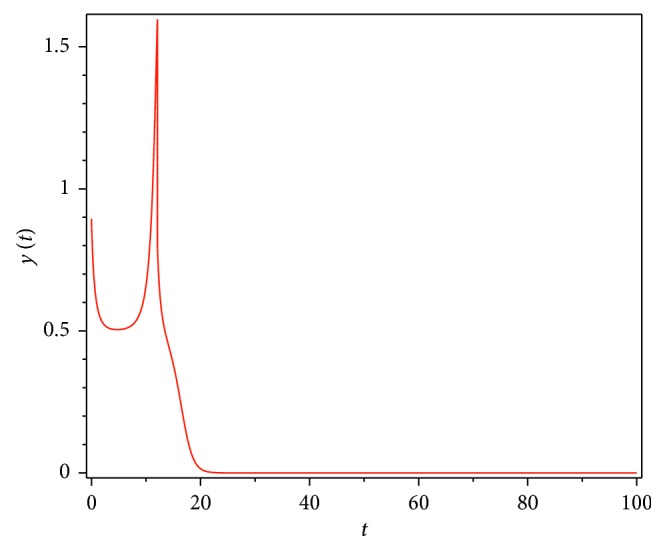
Time series diagram of *y*(*t*) of system (3) with *p*=0.65.

Then, we consider the state-dependent pulse control strategy in system (3). First, we take more moderate preventive and therapeutic measures, and let *p*=0.496, *q*=0.5, then system (3) has a homoclinic cycle composed of the unstable manifold (Γ_*A*_), the stable manifold (Γ_*B*_), and the pulse straight line (Figure 9, the initial value is *S*_0_=0.15  and  *I*_0_=0.895). And from the time series diagrams, we can see that *x* and *y* show periodic oscillations over time (Figures 10 and 11). If we maintain a certain intensity of treatment (fix parameter *q*=0.5) and reduce the intensity of prevention, for example, let *p*=0.3, by Theorem 2, the order-1 homoclinic cycle disappears and bifurcates a order-1 periodic solution, which is shown in Figures 12–14. And if we take more stringent preventive measures which means a larger vaccination rate, for example, let *p*=0.65, then the disease will become extinct, which is shown in Figures 15–17.

## 6. Conclusion

In this paper, a different strategy from tradition, i.e., the state-dependent pulse vaccination and therapeutic strategy, is considered in the control of the disease. A pulse system is built to model this process based on an SI ordinary differential equation model. By using the theory of semicontinuous dynamic system, the dynamics of the pulse system is analyzed. Our results show the pulse system exhibits rich dynamics; for example, the system has a unique order-1 homoclinic cycle, and by choosing *p* as the control parameter, we prove that when *p* changes, the order-1 homoclinic cycle disappears and bifurcates an orbitally asymptotical stable order-1 periodic solution. However, it should be pointed out here that, in this work, we focused on the theoretical framework and realistic parameters can be incorporated into our model. State-dependent impulsive vaccination strategy may be used a supplementary control measure besides routine vaccination, or it may be used in the situation when vaccine stockpile is limited (for example, the yellow fever outbreaks in Nigeria and Congo in 2017 [52]). The realistic approach in childhood infection and other infections will be conducted in future work.

## Data Availability

All data are hypothetical to verify the theoretical results of this study.
